# Potent anti-tumor activity of a syringolin analog in multiple myeloma: a dual inhibitor of proteasome activity targeting β2 and β5 subunits

**DOI:** 10.18632/oncotarget.24160

**Published:** 2018-01-11

**Authors:** Takashi Yoshida, Masaki Ri, Takashi Kanamori, Sho Aoki, Reham Ashour, Shiori Kinoshita, Tomoko Narita, Haruhito Totani, Ayako Masaki, Asahi Ito, Shigeru Kusumoto, Takashi Ishida, Hirokazu Komatsu, Shun Kitahata, Takuya Chiba, Satoshi Ichikawa, Shinsuke Iida

**Affiliations:** ^1^ Department of Hematology and Oncology, Nagoya City University Graduate School of Medical Sciences, Nagoya, Japan; ^2^ Center for Research and Education on Drug Discovery, Faculty of Pharmaceutical Sciences, Hokkaido University, Hokkaido, Japan

**Keywords:** multiple myeloma, syringolin analog, dual inhibitor, proteasome, bortezomib resistance

## Abstract

Proteasome inhibitors (PI), mainly targeting the β5 subunit of the 20S proteasome, are widely used in the treatment of multiple myeloma (MM). However, PI resistance remains an unresolved problem in the therapy of relapsed and refractory MM. To develop a new PI that targets other proteasome subunits, we examined the anti-MM activity of a novel syringolin analog, syringolog-1, which inhibits the activity of both the β5 and β2 subunits. Syringolog-1 exhibited marked cytotoxicity against various MM cell lines and anti-tumor activity towards bortezomib (Btz)-resistant MM cells through the dual inhibition of chymotrypsin-like (β5 subunit) and trypsin-like (β2 subunit) activities. MM cells, including Btz-resistant cells, showed elevated CHOP and NOXA expression after syringolog-1 treatment, indicating the induction of excessive endoplasmic reticulum stress during syringolog-1 treatment. Similar activities of syringolog-1 were also observed in freshly prepared MM cells derived from patients. To clarify the anti-tumor mechanism of dual inhibition of both the β5 and β2 subunits of the proteasome, *PSMB5* and *PSMB7* were co-inhibited in MM cells. This resulted in increased apoptosis of MM cells accompanied by accumulation of ubiquitinated proteins compared to inhibition of either *PSMB7* or *PSMB5* alone, indicating an enhanced effect by double inhibition of β2 and β5 activities. In conclusion, this syringolin analog, a dual inhibitor of proteasome β2 and β5 activities, exhibited potent anti-tumor effects on MM cells and may be useful for overcoming Btz-resistance in the treatment of MM.

## INTRODUCTION

Multiple myeloma (MM) is a mature B cell neoplasm characterized by abundant secretion of monoclonal immunoglobulin and other unfavorable symptoms represented as myeloma-defining events [1]. Several novel agents have been developed and introduced to treat this incurable disease. Among them, the proteasome inhibitor bortezomib (Btz) was the first proteasome inhibitor approved for clinical use and is widely used in the treatment of MM including newly diagnosed and relapsed/refractory cases [2]. Recently, novel proteasome inhibitors have been introduced and are expected to be effective and less toxic in the treatment of MM [3, 4] .

The 26S proteasome is a catalytic complex composed of the 20S proteasome and 19S regulatory subunit and promotes the proteolysis of ubiquitinated proteins to be degraded [5]. The 20S proteasome has three functional activities: caspase-like (C-L, β1 subunit), trypsin-like (T-L, β2 subunit), and chymotrypsin-like (CT-L, β5 subunit) activities. The anti-tumor effect of Btz and novel proteasome inhibitors in clinical use mainly depend on the inhibition of CT-L activity [5]. In fact, two reversible proteasome inhibitors, Btz and ixazomib, markedly inhibit CT-L activity and weakly inhibit C-L activity [6, 8]. Carfilzomib, an irreversible proteasome inhibitor, specifically targets CT-L activity [7]. Inhibition of CT-L activity is considered a key strategy for treating MM cells. However, several studies have proposed that the mechanism of action of Btz resistance is mainly associated with activity of the β5 subunit, such as mutations in *PSMB5* (β5 subunit coding gene) [9, 10] or upregulated expression of proteasome subunits [11–14]. Specifically, inhibiting the activity of the β5 subunit would not provide a sufficient anti-tumor effect in MM cases showing Btz resistance. Therefore, targeting proteins other than the β5 subunit is considered a novel strategy for inducing cell death in MM cells that are insensitive to β5 inhibition.

Syringolin A is a novel proteasome inhibitor extracted from *Pseudomonas syringae* pv *Syringae,* and belongs to the syrbactin class of proteasome inhibitors [15]. Although this compound irreversibly inhibits 20S proteasome activity, it has poor cell membrane permeability because of its hydrophilicity, necessitating the administration of high doses at the micromolar level to effectively inhibit proteasome activity [16]. Therefore, we developed a new syringolin analog showing strong and stable proteasome inhibition and improved the biological activity of this compound. We recently developed several novel syringolin analogs exhibiting remarkable proteasome inhibition with favorable cell permeability [17, 18] and potent proteasome inhibition at the nanomolar level in human tumor cells.

**Figure 1:**
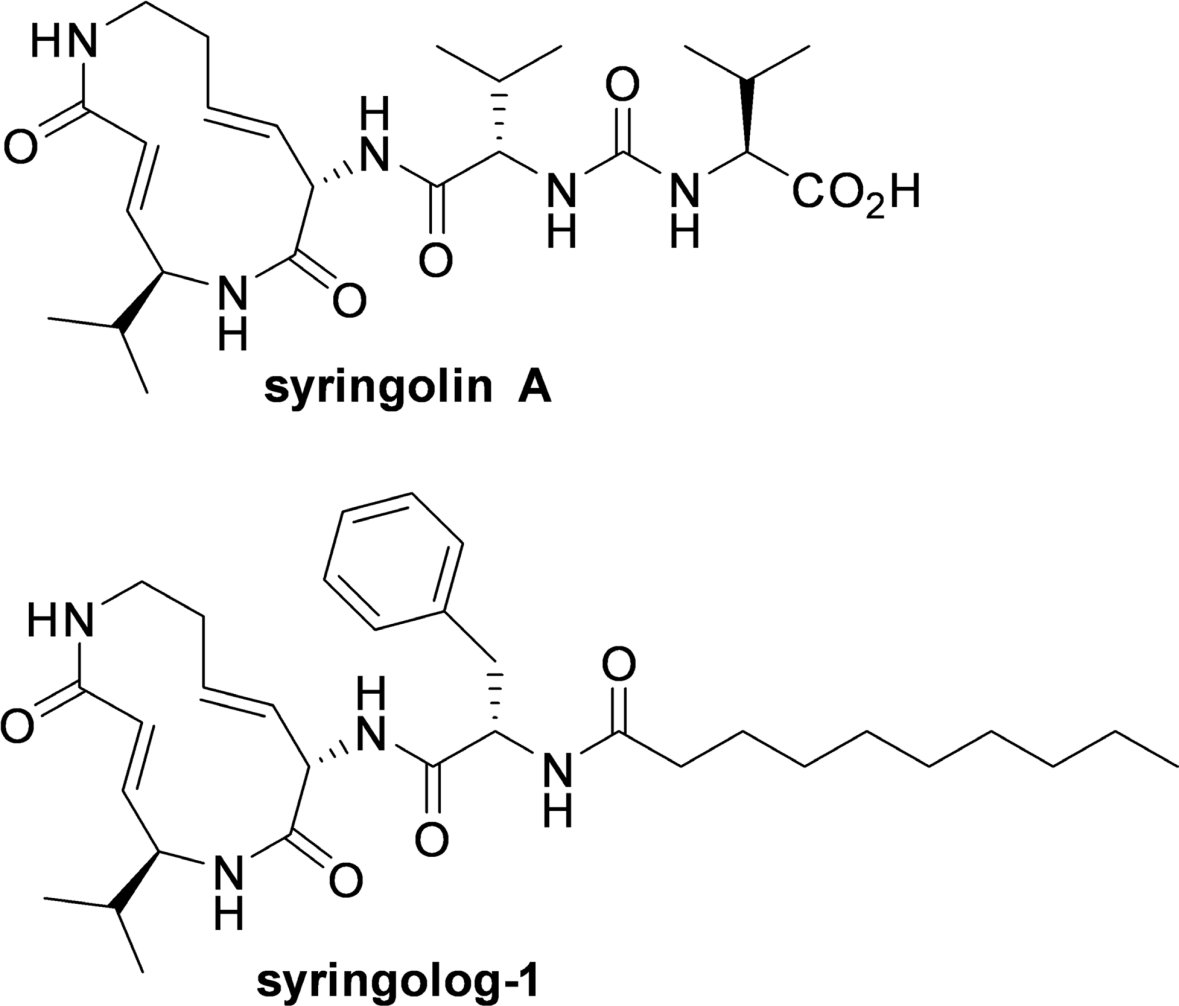
Structure of syringolin A and its synthetic analog, syringolog-1 The structural formula of syringolog-1 is indicated.

Here, we examined the anti-MM effect of a novel syringolin compound named as syringolog-1 (Figure 1), which inhibits both CT-L and T-L activities in MM cells, and found that dual inhibition of the CT-L and T-L activities of the 20S proteasome was a potent treatment strategy for MM, including Btz-resistant cases.

## RESULTS

### Syringolog-1 exhibits an anti-tumor effect on bortezomib-resistant MM cells through dual inhibition of chymotrypsin-like and trypsin-like activities

A total of 10 cell lines, including 4 MM cells, 3 lymphoma cells, and 3 cells harboring Btz resistance, were utilized to evaluate the growth inhibitory effect of syringolog-1. The mean IC_50_ values of syringolog-1 on these cells were approximately 10 nM, with most values lower than the IC_50_ value of Btz (Table 1). Both MM cells and lymphoma cells showed a remarkable decrease in cell viability upon treatment with around 10 nM syringolog-1.

**Table 1:** The IC_50_ of MM, lymphoma, and bortezomib resistant cell lines in each drug

		Syringolog-1		Bortezomib	
		IC_50_	IC_50_	IC_50_	IC_50_
Cell type	Cell name	(nM)	ratio ^*^	(nM)	ratio ^*^
*MM*					
	U266	5.9		15	
	RPMI8226	6.4		20.1	
	KMS-11	5.7		5.4	
	OPM-2	1.3		2.8	
*Lymphoma*					
	MT-4	1.2		8.3	
	Hut-78	7.6		22.5	
	Jurkat	5.7		37.4	
*Btz resistan*t					
	KMS-11/Btz	17.4	3.1	240	44.4
	OPM-2/Btz	5.1	3.9	293	104.6
	MT-4/Btz	6.4	5.3	69.5	8.4

^*^ Comparison with parental cell line. Btz, bortezomib.

After the incubation with each drug for 72hrs, the IC_50_ values were determined.

Three Btz-resistant cell lines, KMS-11/Btz, OPM-2/Btz, and MT-4/Btz, showed a 3.1- (IC_50_ = 17.4 nM), 3.9- (IC_50_ = 5.1 nM), and 5.3 (IC_50_ = 6.4 nM)-fold higher resistance to syringolog-1 compared to their parental cells, KMS-11 (IC_50_ = 5.7 nM), OPM-2 (IC_50_ = 1.3 nM), and MT-4 (IC_50_ = 1.2 nM). This resistance to syringolog-1 was significantly lower than the resistance to Btz, which was 44.4-fold in KMS11/Btz, 104.6-fold in OPM2/Btz, and 8.4-fold in MT-4/Btz (Table 1).

**Figure 2:**
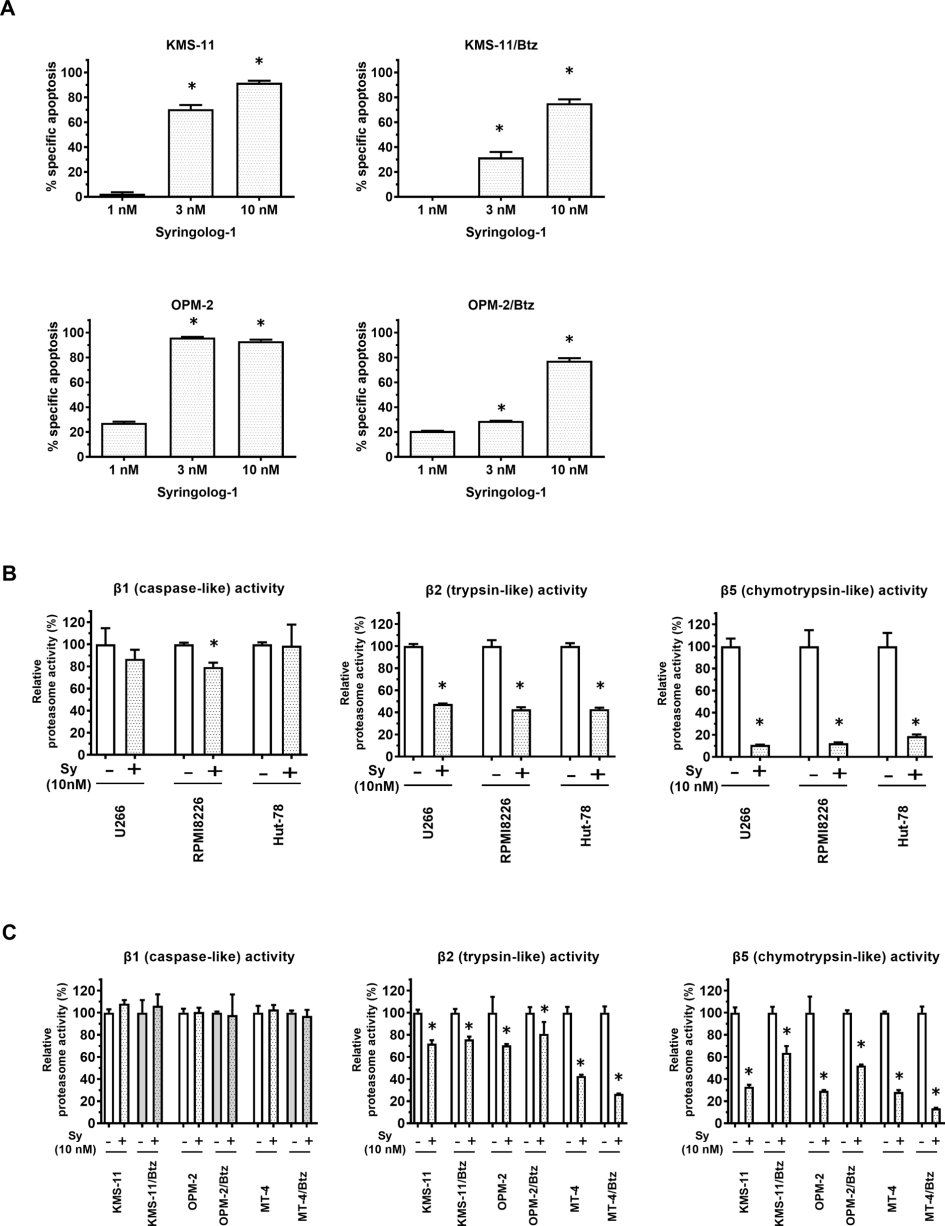
Induction of apoptosis and alteration of proteasome activities in multiple myeloma cells treated with syringolog-1 (**A**) Percent specific apoptosis in multiple myeloma (MM) cell lines and bortezomib (Btz)-resistant cell lines was detected after incubation with 10 nM of syringolog-1 for 16 h. ^*^ represents statistically significant (*p* < 0.05) by Dunnett’s post*-test*. (**B**) MM cell lines were subjected to the analysis of proteasome activities after incubation with 10 nM syringolog-1 (Sy) for 6 h. (**C**) Btz-resistant MM cell lines and their parental cells were similarly analyzed of proteasome activities. ^*^ represents statistically significant by Student’s *t*-test (*p* < 0.05).

Upon measuring apoptosis induced by syringolog-1 treatment, two MM cell lines, KMS-11 and OPM-2, showed significant levels of apoptosis in a dose-dependent manner (Figure 2A, left). A similar result was observed in the corresponding Btz-resistant cell lines (Figure 2A, right).

To evaluate syringolog-1-induced proteasome inhibition, alterations in 20S proteasome activities were measured upon syringolog-1 treatment in various cell lines, including Btz-resistant cells. Similar to Btz treatment, most cells, including MM and lymphoma cells, showed a remarkable reduction in CT-L activity by at least 80% and mild or no reduction in C-L activity (Figure 2B–2C: left, right). Unlike with Btz, a moderate to mild reduction in T-L activity of approximately 20-50% was observed in most cells tested; this reduction was also observed in Btz-resistant cells (Figure 2B–2C: middle), suggesting that inhibition of T-L activity was not affected by Btz resistance during syringolog-1 treatment.

**Figure 3:**
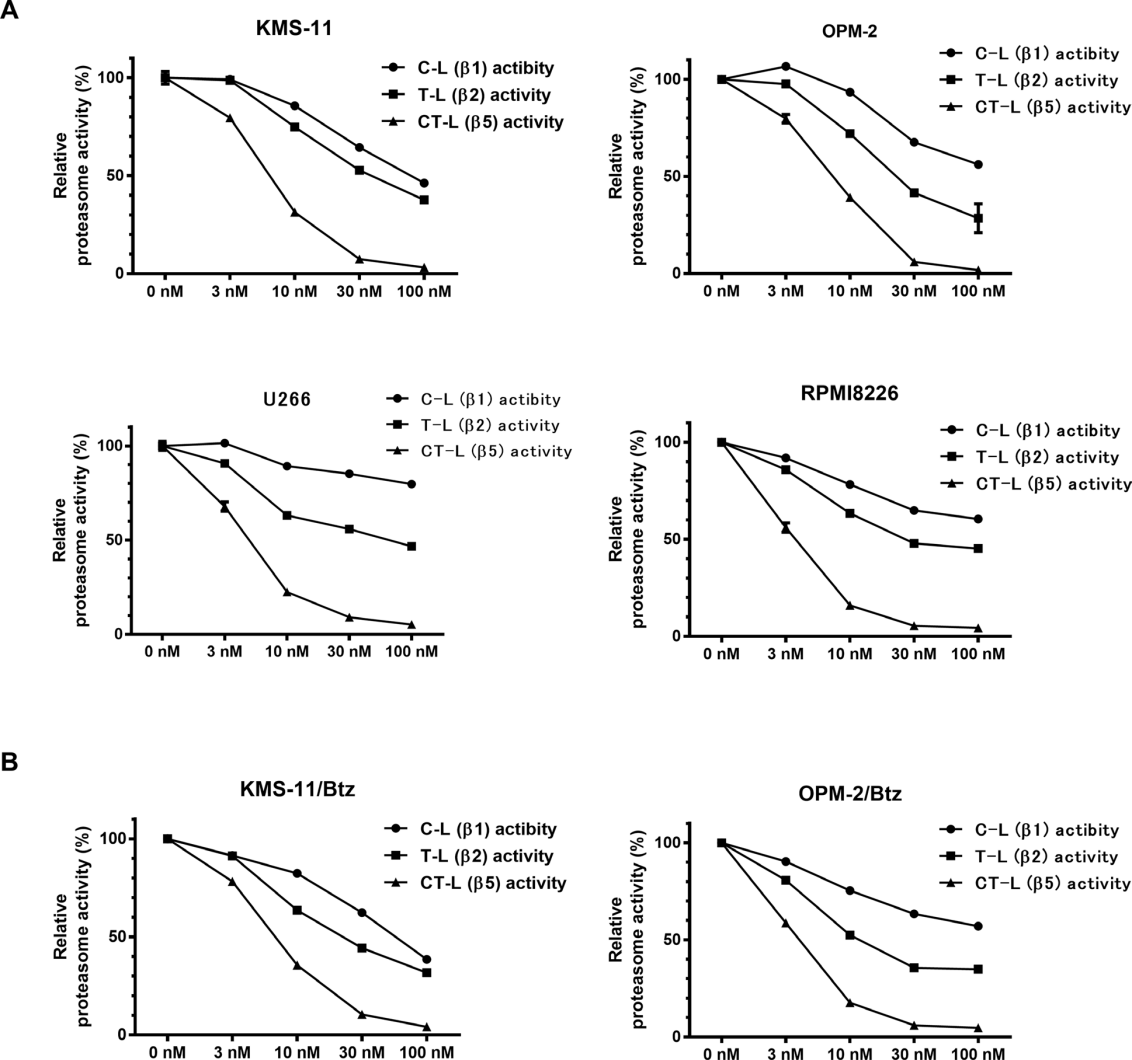
Alteration of proteasome activities in multiple myeloma cells treated with various concentration of syringolog-1 (**A**) Six MM cells were subjected to the analysis of proteasome activities after incubation with indicated dose of syringolog-1 for 6 h. Each value was calculated as the mean value of triplicate experiments. (**B**) Two Btz-resistant cell lines were analyzed similarly.

Next, we evaluated the inhibitory effect of various concentrations of syringolog-1 on the above three activities. As shown in Figure 3A, 4 MM cell lines, KMS-11, OPM-2, U266, and RPMI8226, showed a remarkable reduction in CT-L activity, moderate to mild reduction in T-L activity, and mild reduction in C-L activity in a dose-dependent manner. Similar reductions in the activity of each proteasome were observed in the two Btz-resistant cell lines (Figure 3B).

**Figure 4:**
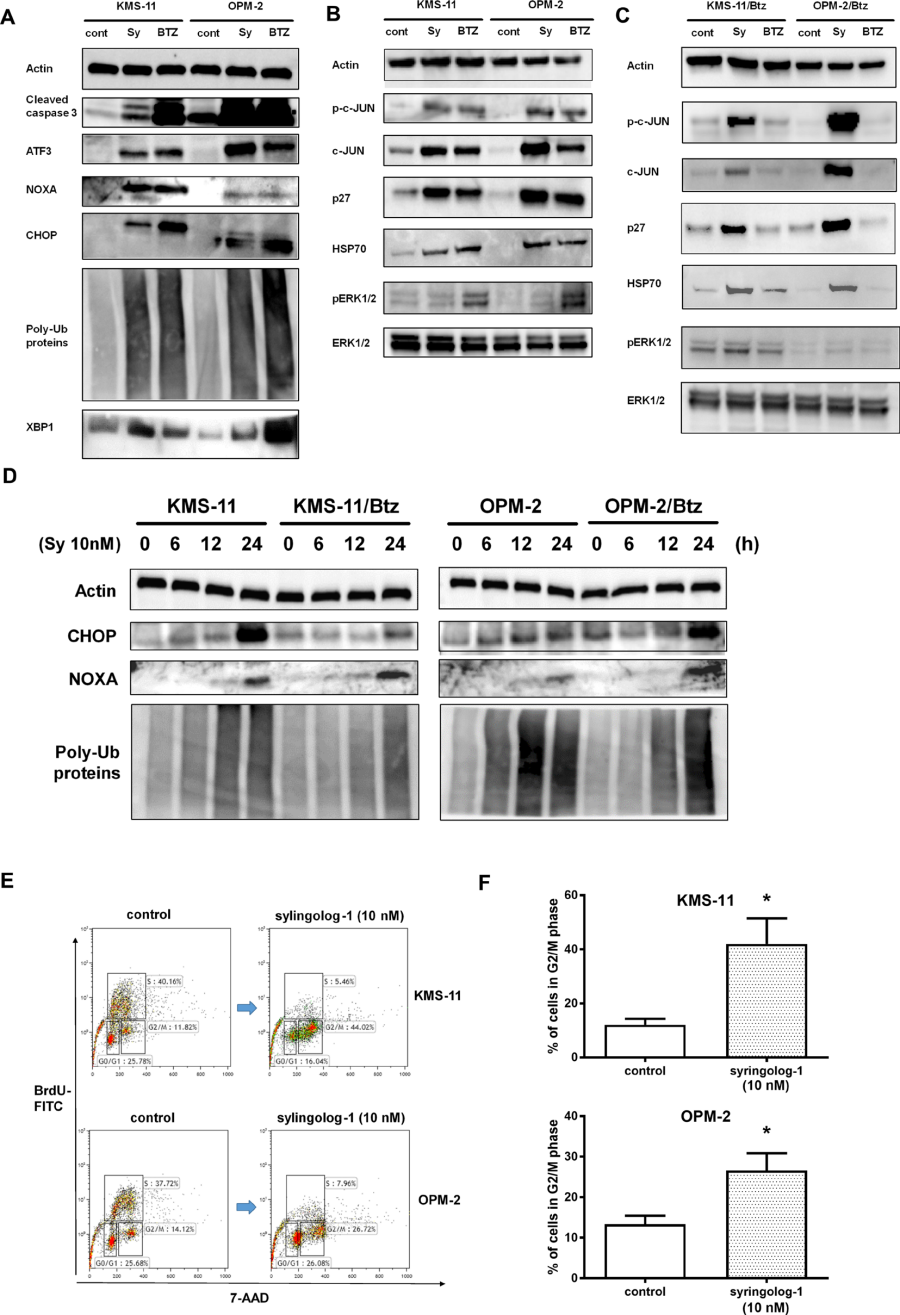
Evaluation of ubiquitin-proteasome, ER stress, and apoptosis-related pathways after syringolog-1 treatment by immunoblot analysis (**A**–**C)** Two multiple myeloma (MM) cells, KMS-11 and OPM-2, and Btz-resistant cells, KMS-11/Btz and OPN-2/Btz, were incubated with 10 nM of syringolog-1 (Sy) or bortezomib (Btz) for 16 h, and subjected to the analysis of changes in ubiquitin-proteasome, ER stress, and apoptosis-related pathways. (**D**) Btz-resistant MM cell lines and their parental lines were similarly evaluated. (**E**) Analysis of cell cycle distribution before and after the 10 nM of syringolog-1 treatment for 16 h. A representative case of three independent experiments. (**F**) Comparison of the ratio in G2/M phase between the control and the treatment by 10 nM of syringolog-1 for 16 h in two MM cells. ^*^ represents statistically significant by Student’s *t*-test (*p* < 0.05).

### Alteration of ubiquitin-proteasome, endoplasmic reticulum stress, and apoptosis-related pathways during syringolog-1 treatment

To clarify the mechanism of action underlying syringolog-1-induced cell death, alterations in pathways associated with the unfolded protein response (UPR) and endoplasmic reticulum (ER) stress were assessed. After syringolog-1 treatment, two MM cell lines showed increased expression of CHOP, ATF3, and XBP1 and accumulation of poly-ubiquitin proteins related to UPR and ER stress, as well as upregulation of NOXA and activation of cleaved caspase 3 (Figure 4A). Similar results were observed in two Btz-resistant cell lines tested in the same manner (Figure 4D). Phosphorylation of c-Jun and ERK1/2, and activation of p27 and Hsp70, considered as indicators of the cell stress response, were observed upon syringolog-1 treatment (Figure 4B). These results were also observed following Btz treatment. Two Btz-resistant cell lines were similarly tested with parental cells. As shown in Figure 4C, Btz resistant cells showed minimal changes in phosphorylation and activation of the above substrate after bortezomib treatment. However, following treatment with syringolog-1, these cells showed the same phosphorylation and activation levels as treatment with each substrate in parental cells.

To examine the effect of syringolog-1 on the cell cycle distribution, the cell cycle was analyzed before and after syringolog-1 treatment. As shown in Figure 4E and 4F, accumulation of the G2/M phase was observed in both MM cells after syringolog-1 treatment.

### Evaluation of syringolog-1-induced anti-tumor effects in primary MM cells

**Figure 5:**
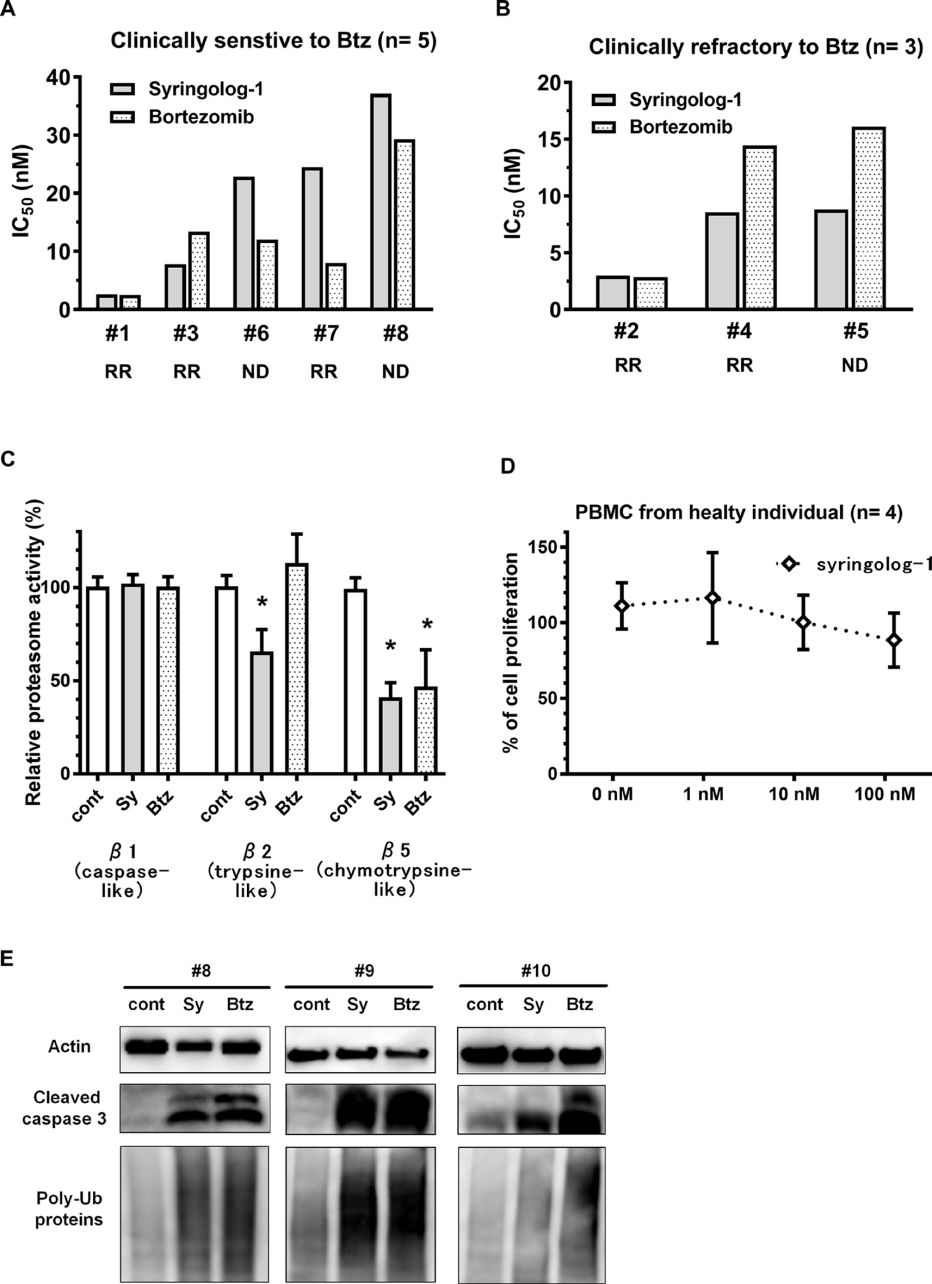
Evaluation of syringolog-1 activity in primary MM cells from patients (**A**–**B**) Cytotoxicity of syringolog-1 (Sy) or bortezomib (Btz) was tested on 8 primary MM cells. IC_50_ values of Sy on each primary MM cell were calculated from the triplicate experiments. A. 5 cases clinically sensitive to Btz-based therapy. (B) 3 cases clinically refractory to Btz-based therapy. RR: relapsed and or refractory, ND: newly diagnosed. (**C**) 20S proteasome activities in 8 primary MM cells were evaluated after incubation with or without 10 nM of Sy or Btz for 6 h. (**D**) Four peripheral blood mononuclear cells from healthy individuals were analyzed for cytotoxic effect induced by Sy treatment at the indicated dose for 48 h. (**E**) Accumulation of poly-ubiquitinated proteins and expression of cleaved caspase-3 were evaluated in 3 primary MM cell lines after incubation with 10 nM of syringolog-1 or Btz for 16 h. ^*^ represents statistically significant (*p* < 0.05) by Dunnett’s post*-test*.

Eight primary MM samples derived from patients were utilized to examine syringolog-1-induced anti-tumor effects using the same methods used for the cell lines described above. The median IC_50_ values of syringolog-1 and Btz on 8 primary MM cells were 8 and 13 nM, respectively. Among the 5 primary MM cells showing clinical sensitivity to Btz-based therapy, most cases showed IC_50_ values of 15 nM of lower following Btz treatment (Figure 5A). These cells showed equal or slightly higher IC_50_ values after syringolog-1 treatment. In the 3 cases with clinical refractory to Btz-based therapy, two cases showed lower IC_50_ values of 8.5 and 8.8 nM after syringolog-1 treatment compared to Btz with values of 14.4 and 16.1 nM (Figure 5B). One sample showed nearly equal IC_50_ values of 2.9 nM after syringolog-1 treatment and 2.8 nM after Btz treatment. Regarding inhibition of 20S proteasome activities, Btz inhibited CT-L activity, whereas syringolog-1 showed not only remarkable inhibition of CT-L but also moderate inhibition of T-L activity (Figure 5C). To evaluate the cytotoxic effect of syringolog-1, peripheral blood mononuclear cells (PBMCs) from healthy individuals were incubated with various concentrations of syringolog-1. As shown in Figure 5D, no or a low cytotoxic effect was observed in PBMCs at concentrations below 100 nM.

In immunoblot analysis, three primary MM cell lines accumulated poly-ubiquitin proteins and cleaved caspase-3 upon syringolog-1 treatment (Figure 5E).

### Effect of dual inhibition of PSMB5 and PSMB7 on tumor cells

**Figure 6:**
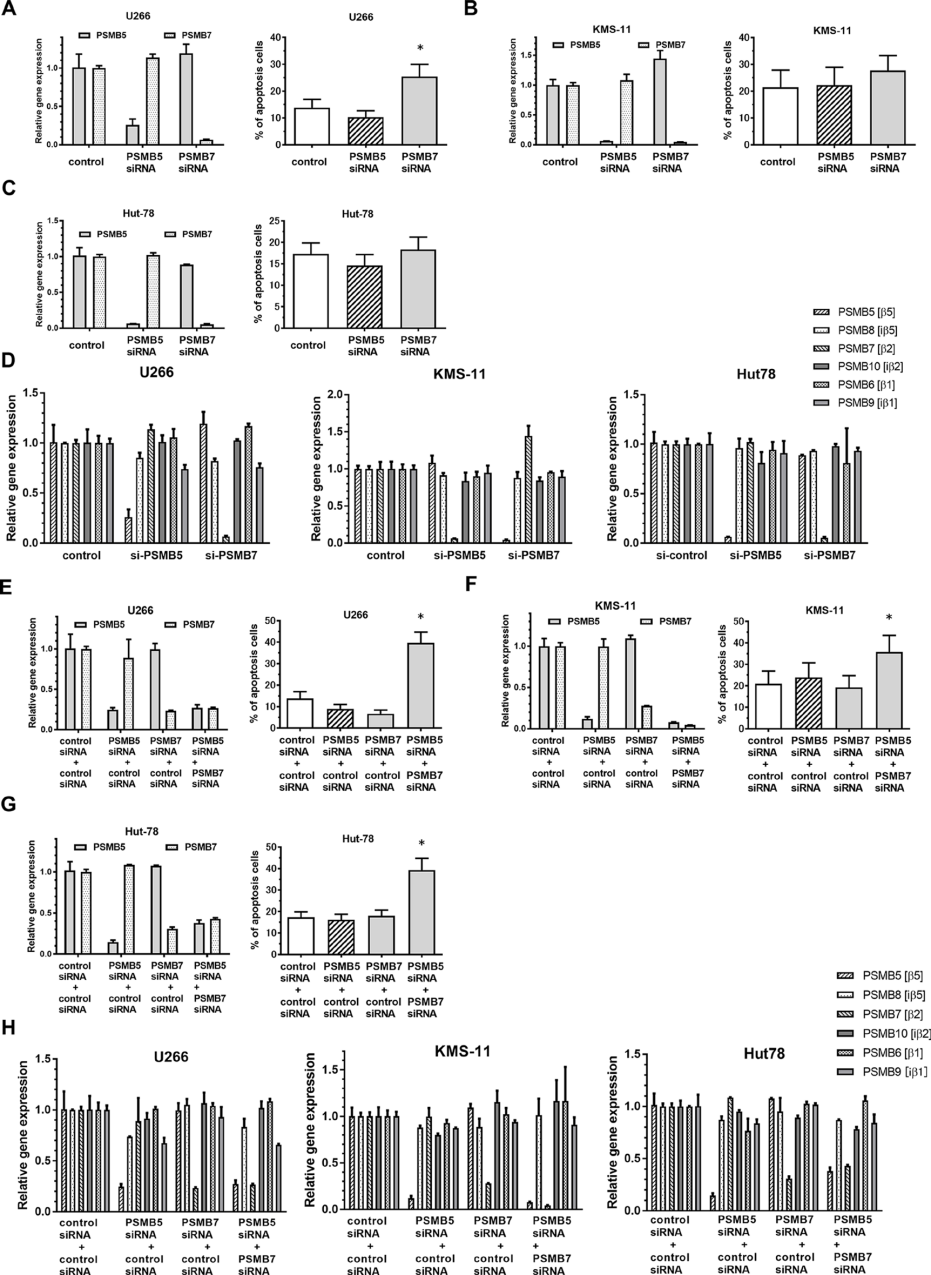
Apoptosis induction and altered expression of ubiquitinated proteins by co-inhibition of expression of PSMB5 and PSMB7 in tumor cells (**A**–**C**) Two MM cell lines, U266 and KMS-11, and a lymphoma cell line, Hut78, were subjected to the inhibition of either *PSMB5* or *PSMB7* by transfection with specific siRNA, and apoptosis induction was subsequently analyzed. (**D**) Alteration of six genes, coding proteasomes, were evaluated at the expression level after transfection of siRNA targeting *PSMB5* or *PSMB7* in tumor cell lines. (**E**–**G**) Three cell lines were subjected to co-inhibition of *PSMB5* and *PSMB7* using suboptimal doses of each specific siRNA. (**H**) Alteration of six genes, coding proteasomes, were evaluated at the expression level after transfection of siRNA targeting *PSMB5* and or *PSMB7* in tumor cell lines. ^*^ represents statistically significant (*p* < 0.05) by Dunnett’s pos*t*-test.

Unlike Btz, syringolog-1 is thought to target both CT-L and T-L activities, which may contribute to its enhanced anti-tumor activity in MM cells. To elucidate the significance of dually suppressing CT-L and T-L 20S proteasome activities, the expression of *PSMB5* and PSMB7, encoding the β5 and β2 subunits, respectively, was suppressed in tumor cells and then cell viability was evaluated. In U266 cells, when gene expression of either *PSMB5* or *PSMB7* was silenced by siRNA (Figure 6A), *PSMB7*-knockdown cells showed a higher rate of apoptosis estimated as 25.4% compared to *PSMB5* -knockdown and control cells, which exhibited rates of 10.3% and 13.8% apoptosis, respectively. Similarly, greater apoptosis in *PSMB7*-knockdown cells was observed in two other cell lines, KMS-11 and Hut78 (Figure 6B–6C). In the analysis of gene expression in whole proteasomes including *PSMB5* (β5), *PSMB8* (iβ5), *PSMB7* (β2), *PSMB10* (iβ2), *PSMB6* (β1), and *PSMB9* (iβ1), these genes alternated or showed only slight in their expression following silencing of *PSMB5* or *PSMB7* in three tumor cells (Figure 6D).

Next, dual inhibition of *PSMB5* and *PSMB7* were performed in tumor cells using a combination of each specific siRNA at a suboptimal dose. As shown in Figure 6E, single inhibition of either *PSMB5* or *PSMB7* at suboptimal concentrations showed decreased the progression of apoptosis by 9.0% and 6.7%, respectively. However, the combination of these suboptimal knockdown conditions enhanced apoptosis by approximately 39.6% compared to each single inhibition (Figure 6E). A similar enhancement of apoptosis was also confirmed in the cell lines KMS-11 and Hut78, which were tested in the same manner (Figure 6F–6G). In the analysis of gene expression in whole proteasomes, the expression of the 6 genes coding each proteasome was not altered or was only slightly changed after combined transfection with a suboptimal dose of the two siRNAs targeting *PSMB5* and *PSMB7* (Figure 6H) in three tumor cell lines.

**Figure 7:**
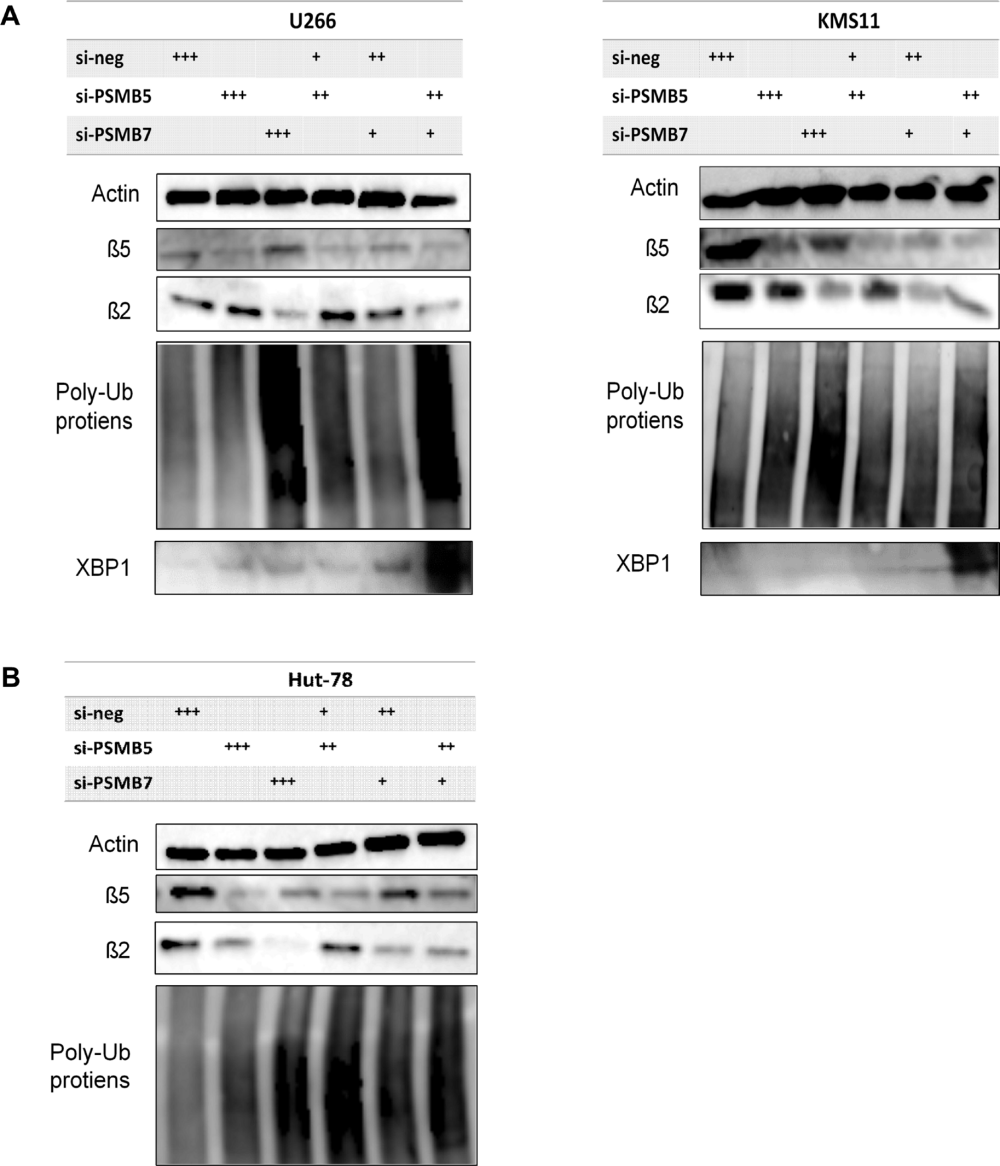
Altered expression of ubiquitinated proteins by co-inhibition of expression of PSMB5 and PSMB7 in tumor cells (**A**–**B**) Altered expression of β5, β2, and poly-ubiquitinated proteins was evaluated after single inhibition of *PSMB5* or *PSMB7* in two MM cells. Similarly, altered expression of these proteins was tested after co-inhibition of *PSMB5* and *PSMB7* using a suboptimal dose of each specific siRNA. ^+^, 25 nM; ^++^, 150 nM; ^+++^; 175 nM. C. Similarly, Hut 78 cell was tested. ^*^ represents statistically significant (*p* < 0.05) by Dunnett’s pos*t*-test compared to control.

In immunoblot analysis, knockdown of either *PSMB5* or *PSMB7* fully suppressed the β5 and β2 subunits in U266 cells (Figure 7A, left). Regarding the accumulation of poly-ubiquitinated proteins, *PSMB7* silencing induced greater accumulation of poly ubiquitinated proteins than *PSMB5* silencing (Figure 7A, left). When evaluating the dual inhibition of *PSMB5* and *PSMB7*, combining suboptimal inhibition conditions of the β5 and β2 subunits triggered more potent accumulation of poly-ubiquitinated proteins and over-expression of XBP1 than suboptimal suppression of either the β5 or β2 subunit alone, indicating excess ER stress (Figure7A, left). The same results of excessive ER stress were observed in KMS-11 cells with dual inhibition of *PSMB5* and *PSMB7* (Figure 7A, right). Similarly, facilitated accumulation of poly-ubiquitinated proteins was confirmed in Hut78 cells upon inhibiting both the β5 and β2 subunits (Figure 7B).

Taken together, these results demonstrate that dually inhibiting the β2 and β5 subunits of the 20S proteasome had an additive effect on cytotoxicity compared to inhibiting either of these subunits alone.

### *In vivo* anti-tumor activity of syringolog-1 alone in a human MM xenograft model

**Figure 8:**
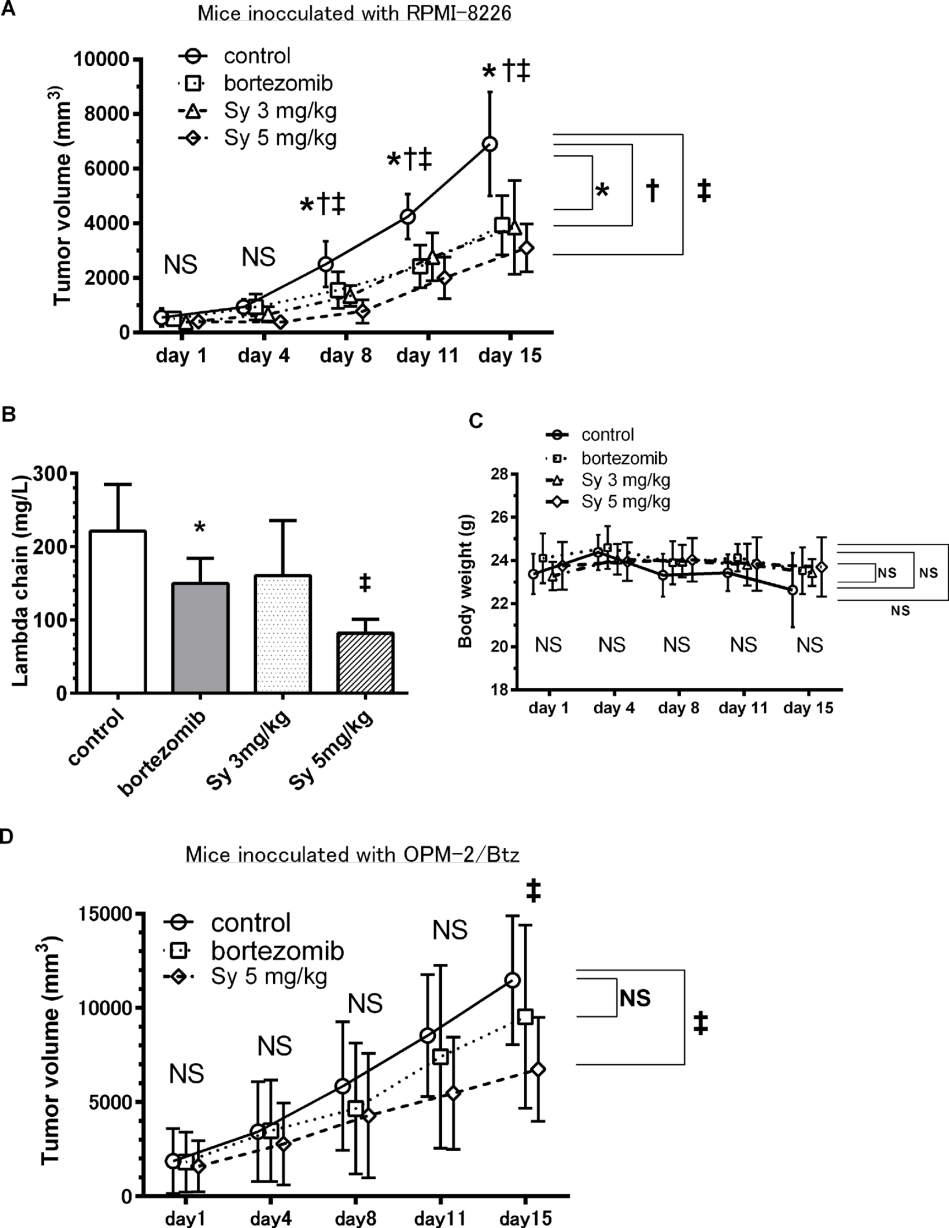
Syringolog-1 exerts anti-tumor activity in an *in vivo* MM model (**A**) Average tumor volume in untreated mice (normal saline) and mice treated with either syringolog-1 (Sy) at 3 and 5 mg/kg, or bortezomib (Btz) at 1 mg/kg. (**B**) Average value of free light chains in plasma was measured in each treatment group on day 16. (**C**) Changes in body weight in each treatment group are shown. (**D**) Average tumor volume in mice inoculated with OPM-2//BTZ cells. Mice were divided into three groups, untreated, treated with either Sy at 5 mg/kg, or Btz at 1 mg/kg. ^*^, ^†^, ^‡^; represents statistically significant (*p* < 0.05) by Dunnett’s post*-test*, compared to control.

To evaluate the *in vivo* efficacy of syringolog-1 in MM cells, SCID mice subcutaneously inoculated with RPMI-8226 were administered syringolog-1 intraperitoneally twice weekly at either 3 or 5 mg/kg. Syringolog-1 treatment showed robust anti-tumor activity, resulting in significantly smaller tumor volumes compared to control tumors on day 8, 11, and 15. This activity was similar to the effect of BTZ administration (Figure 8A). No obvious difference in the tumor inhibitory effect was observed upon 3 or 5 mg/kg injection of syringolog-1.

In the analysis of serumfree light chain expressed in inoculated MM cells, the mean value of the lambda light chain in the group with 3 mg/kg of treatment was generally lower than the levels in the control group, and mean value of the lambda light chain in the group treated with 5 mg/kg of syringolog-1 was significantly lower than that in the group with Btz treatment and controls (Figure 8B).

Changes in body weight occurred in the control group, perhaps due to the tumor burden (Figure 8C). No mice showed visible changes in appearance that would indicate toxicity.

Finally, to evaluate the anti-MM activity of syringolog-1 against Btz-resistant MM cells, NOD SCID mice subcutaneously inoculated with Btz-resistant MM cells, OPM-2/Btz were administered with syringolog-1 intraperitoneally, twice weekly, at 5 mg/kg. Syringolog-1 treatment showed significant anti-tumor activity, resulting in smaller tumor volumes compared to control tumors or BTZ administration on day 15 (Figure 8D).

## DISCUSSION

In this study, we developed a new syringolin compound, syringolog-1, and showed its ability to dually inhibit both CT-L and T-L activities. We revealed several features of this compound: (1) marked cytotoxicity of syringolog-1 treatment in Btz-resistant cells through dual inhibition of CT-L and T-L activities, (2) accumulated poly-ubiquitinated proteins and subsequent ER stress in Btz-resistant cells upon syringolog-1 treatment, (3) triggering of progressed apoptosis by co-inhibition of the β5 and β2 subunits compared to that by inhibition of individual subunits, and (4) anti-myeloma effect of syringolog-1 treatment without any marked side effects in a murine MM model.

In our study, among the 8 primary MM cases tested, 3 cases showed lower sensitivity to syringolog-1 treatment than Btz, represented by slightly higher IC_50_ values. Although the reason for this low sensitivity in syringolog-1 was unclear, these 3 cases were derived from clinically Btz-sensitive cases, and therefore may have good sensitivity in inhibiting proteasomes β5 and β1 by Btz treatment than inhibition of β5 and β2 by syringolog-1 treatment. Further analysis of a larger number of cases including both Btz-sensitive and refractory cases are needed to evaluate the difference in sensitivity between the two proteasome inhibitors.

As seen in several preclinical studies [19, 20] , the therapeutic potential of proteasome inhibitors is considered to depend not on the predominant inhibition of a single proteasome activity, such as CT-L activity, but on the multiple inhibition of proteasome activities, such as co-inhibition of CT-L and C-L observed in Btz. Carfilzomib (Cfz), a specific and irreversible inhibitor of CT-L activity, was expected to be capable of treating Btz-resistant MM cases; however, it showed decreased clinical efficacy in Btz-resistant cases in the FOCUS study, which targeted Btz- and lenalidomide-resistant MM cases [21, 22] . Cfz mainly improves clinical treatment in patients with MM who received insufficient Btz administration because of the intolerable side effects of the agent, such as severe peripheral neuropathy and gastrointestinal disorders. In addition, Cfz was shown to have a clinical benefit in the treatment of relapsed and refractory (RR) MM cases when combined with lenalidomide plus dexamethasone treatment [4].

Cfz now has potent anti-tumor effects on RR MM cases when this agent is administered at an elevated dose of 20/56 mg/m^2^ [23] . In this administration setting, Cfz has been shown to lose its selectivity of proteasome inhibition and co-inhibit CT-L and T-L activity [24]. In the current study, selective suppression of the β5 subunit induced low apoptosis levels in all 3 cell lines tested, including MM and lymphoma cells, compared to the higher levels of apoptosis observed upon dual inhibition of the β5 and β2 subunits. This result suggests the limited anti-tumor activity of inhibiting a single proteasome activity and the critical role of targeting multiple proteasome activities during MM treatment. To develop an effective method for inhibiting the proteasome to treat MM, the choice of which proteasome activities to co-inhibit should be based on the status of proteasome activity in each MM cell line, which can be accomplished by studying biomarkers using specimens derived from each MM patient, such as tumor cells and serum/plasma from peripheral blood [25].

Recent studies of the direct mechanisms of Btz resistance were derived analyzed various tumor cell lines, including MM [10, 11], lymphoma [26], leukemia [9, 27], lung cancer [12], and hepatocellular carcinoma [14], with Btz. These studies suggested that Btz resistance is controlled by multiple factors, including alterations in *PSMB5* expression or sequence, adjustment of biosynthesis of new proteins to reduce the ER stress [27], and upregulation of multiple proteasome subunits, such as β2 and β5 subunits, which enables cells to adapt to the selective proteasome inhibition resulting from Btz treatment. The therapeutic potential of targeting T-L activity in addition to inhibiting CT-L activity was proposed by a previous study [28]. In this study, the specific β2 inhibitor LU-102 showed synergistic proteasome inhibition and activated the UPR and cytotoxicity in Btz-resistant MM cells when combined with Btz and Cfz treatment. Although the combination of LU-102 and Btz or Cfz is an attractive treatment strategy, monotherapy with LU-102 showed lower cytotoxicity in Btz-resistant MM cells, suggesting that selective elimination of T-L activity alone is insufficient to have a meaningful anti-tumor effect on Btz-resistant MM cells [28]. From this report, although co-inhibition of *PSMB5* and *PSMB7* on Btz-resistant cells was not tested in our study, dual inhibition of CT-L and T-L activities, rather than single inhibition, is indispensable for triggering excessive ER stress and sequential fatal cell stress in Btz-resistant MM cells. In addition, LU-102 has been reported to have several limitations to its potential clinical use, including the need for a high concentration for anti-tumor activity and poor tolerability because of the high amount of vinyl sulfone in combination with Btz observed in a murine model [28]. In the current study, we identified a new syringolin analog, syringolog-1, and characterized its dual inhibition of CT-L and T-L activity by monotherapy. As previously reported, cells treated with Btz exhibit elevated expression of the β2 and β5 subunits [11, 14, 27] . In addition, inhibiting *PSMB7* was reported to re-sensitize MM cells during Btz treatment [29]. Therefore, this compound is expected to have an anti-tumor effect on Btz-resistant MM cells through the co-inhibition of CT-L and T-L activities and may be clinically useful against Btz-resistant MM cells as a single or combination therapy with other agents, such as Btz and Cfz.

Among the several factors associated with the cell survival and apoptosis, HSP70, a member of the cell-protective chaperon, was elevated during syringolog-1 treatment. This elevation was also observed in Btz treatment [30], which is considered a feedback action of the cells to reduce fatal intracellular stress during proteasome inhibition. Therefore, to maximize the anti-tumor effect of syringolog-1, a combination with a HSP-70 inhibitor may be a therapeutic strategy to repress the compensatory action of cell to avoid lethal conditions [31].

In summary, our results demonstrate that the novel syringolin compound syringolog-1 dually inhibited CT-L and T-L proteasome activities, triggering high ER stress and related apoptosis in Btz-resistant MM cells. Our data suggest that targeting both CT-L and T-L activities can overcome Btz resistance, and therefore a dual inhibitor of the β2 and β5 subunits may be a new treatment option for patients with Btz/Cfz-resistant MM cells, who have poor treatment options for this incurable disease.

## MATERIALS AND METHODS

### Cell culture and reagents

In this study, five human MM cell lines, KMS-11, OPM-2, NOP-1, U266, and RPMI8226; and 3 malignant lymphomas cells, MT-4, Jurkat, and Hut78; were used and cultured in RPMI-1640 medium supplemented with heat-inactivated 10% fetal bovine serum at 37°C in a 5% CO_2_ incubator [25, 32, 33]. Two Btz-resistant MM cells, KMS-11/Btz and OPM-2/Btz, were described previously [10]. Btz was purchased from Wako Pure Chemical Industries (Osaka, Japan). Syringolog-1 was synthesized as previously described [17].

### Isolation of primary MM specimens and peripheral blood mononuclear cell from healthy individual

A total of 8 primary MM cell samples, derived from 3 patients with newly diagnosed (ND) MM and 5 patients with relapsed and or refractory (RR) MM, were tested in the cytotoxic and 20S proteasome assays. Of the 8 patients, 3 patients, including 2 RR and 1 ND cases, were clinically refractory to Btz-based therapy, while the other 5 patients were not refractory to Btz-based therapy. In this study, a patient was defined as clinically refractory to Btz based on the International Myeloma Working Group criteria and defined as follows: 1) RR cases non-responsive or showing progressive disease with Btz-based therapy or within 60 days of the last Btz-based therapy [34], 2) ND cases showing non-responsive to the first Btz-based therapy [1]. In the immunoblot analysis, 3 primary MM samples with sufficient cell numbers for the analysis were selected. These samples include 1 sample from the above 8 samples, and other 2 samples form patients with ND MM additionally tested.

Bone marrow aspiration specimens from patients with MM were collected prior to the first (ND MM case) or second (RR MM case) treatment after written informed consent was obtained at Nagoya City University Hospital. Primary MM cells were isolated from the bone marrow mononuclear cell fraction using anti-CD138 antibody-conjugated magnetic beads with an AutoMACS Pro Separator, an automatic magnetic cell sorting system (Miltenyi Biotec, Bergisch Gladbach, Germany). To minimize the effect of contamination with normal plasma cells, only bone marrow specimens for which clonal proliferation of MM cells was confirmed by both pathological diagnosis and flow cytometric analysis were used in this study. In addition, to standardize the experimental conditions, all bone marrow specimens were immediately purified and assayed. PBMCs were obtained from four healthy individuals using Ficoll-Paque (Pharmacia, Uppsala, Sweden). This study protocol using human samples was approved by the Nagoya City University Hospital Institutional Review Board.

### Cell proliferation, cytotoxicity, and apoptosis assays

Cell proliferation and cytotoxicity assays were performed as described previously [33]. The IC_50_ value of each agent was calculated by GraphPad Prism version 6.05 for Windows (GraphPad Software, La Jolla, CA, USA). Apoptosis of cells exposed to syringolog-1 or Btz for 16 h was evaluated using annexin V-FITC and propidium iodide staining (Medical & Biological Laboratories, Nagoya, Japan). The fraction of positive cells was determined using an FACS Calibur (BD Bioscience, San Jose, CA, USA). To evaluate apoptosis, only Annexin V-FITC-positive cells, indicating the progression of early apoptosis, were used to calculate the specific apoptosis. Specific apoptosis was calculated as 100 × (% induced apoptotic cells - % spontaneous apoptotic cells) / (100 - % spontaneous apoptotic cells).

All expressed values represent the mean value of triplicate experiments and the IC_50_ values were calculated using the mean value for each concentration of agent.

### 20S proteasome activity assays

A total of 5 × 10^5^ cells were incubated with syringolog-1 or bortezomib for 6 h. After washing twice with cold phosphate-buffered saline, the cells were resuspended in 50 mM Tris (pH 7.4) buffer containing 5 mM MgCl_2_ and 0.2 mg/mL digitonin, which permeabilizes the cell membrane without disrupting it. Cells were transferred into 96-well flat-bottom plates at a final concentration of 4 × 10^4^ cells in 160 µL of buffer per well. Next, 40 µL of fluorogenic substrate, Suc-LLVY-amc (Enzo Life Sciences, NY, USA) for CT-L, Boc-LRR-amc for T-L and Boc-LLE-amc for C-L activities was added to each well at a final concentration of 75 µM. After incubation for 3 h at 37°C, fluorescence was measured at an excitation wavelength of 380 nm and emission wavelength of 460 nm. All expressed values represent the average of triplicate experiments.

### Cell cycle analysis

The cell cycle was analyzed with the BrdU Flow Kit (BD Biosciences). Cells were incubated with 10 nM syringolog-1 for 16 h, and then collected and assayed according to the manufacture instructions. Briefly, cells were fixed in 70% cold ethanol for 30 min on ice and incubated with FITC-conjugated anti-BrdU antibody in PBS containing 0.1% bovine serum albumin for 30 min. Cells were washed in PBS, stained with 7-AAD and analyzed on a FACS Calibur to assess cell cycle distribution.

### Immunoblot analysis

MM cell lines and primary MM samples were incubated with syringolog-1 or Btz for the indicated times. Preparation of whole-cell extracts and their analysis was carried out as described previously [26]. Antibodies against ubiquitin, CHOP, ERK1/2, p-ERK1/2, ATF3, XBP1, and actin were purchased from Santa Cruz Biotechnology (Dallas, TX, USA). Antibodies against cleaved caspase 3, HSP70, c-JUN, p-c-JUN, p27, and the β2 proteasome subunit were purchased from Cell Signaling Technology (Danvers, MA, USA). The antibody against caspase 8 was purchased from BD Bioscience (Franklin Lakes, NJ, USA). The antibody against the β5 proteasome subunit was purchased from Abcam (Cambridge, UK).

### RNA interference assays

Small interfering RNA (siRNA) was used to repress the expression of *PSMB5* and *PSMB7* mRNA. PSMB5 siRNA (ID s11354), PSMB7 siRNA (ID s668), and Silencer Select Negative siRNA (ID 4390843) for the non-targeting control were purchased from Applied Biosystems (Foster City, CA, USA). Electroporation was carried out with the aid of a Nucleofector 2b device (Lonza, Basel, Switzerland) according to the manufacturer’s instructions. Briefly, 2 × 10^6^ U266 cells in kit C nucleofector solution containing siRNA were electroporated using the X-005 program, transferred into culture plates, and incubated. Similarly, KMS-11 cells in kit L nucleofector solution with siRNA were electroporated using the X-001 program and Hut-78 cells in kit R nucleofector solution with siRNA were electroporated using the V-001 program.

### Quantitative real-time reverse transcription PCR

Total RNA was extracted from purified MM cells stored at -80°C with RNeasy Mini kits (Qiagen, Hilden, Germany). Reverse transcription and amplification of total RNA was performed using the High capacity RNA-to-cDNA kit (Thermo Fisher Scientific, Waltham, MA, USA). Quantitative PCR was carried out with SYBR Green Gene Expression Assays (Toyobo, Osaka, Japan) and a Step One Plus Real-Time PCR instrument (Applied Biosystems) according to the respective manufacturers’ instructions. Targeted primer sets for real-time PCR (PSMB5, PSMB7, and actin) were purchased from Takara Bio (Shiga, Japan). The values of all samples were determined by calculating the means of duplicate samples, and adjusted to the expression of actin mRNA as an endogenous control.

### Murine xenograft model

Six-week-old male CB-17/Icr-scid Jcl mice were purchased from CLEA Japan (Tokyo, Japan). All *in vivo* experiments were performed in conformity with the UK Coordinating Committee on Cancer Research Guidelines for the Welfare of Animals in Experimental Neoplasia (Second Edition). A quantity of 5 × 10^6^ RPMI8226 cells suspended in 100 µL RPMI-1640 medium with 50% Corning Matrigel Basement Matrix (Corning, Inc., Corning, NY, USA) were inoculated subcutaneously into SCID mice. One day before tumor inoculation, 50 µL of rabbit anti-asialo-GM1 (Wako Pure Chemical Industries, Osaka, Japan) was administered intraperitoneally. Ten days after tumor inoculation, the tumor-bearing mice were divided into four groups of six mice each so that the mean tumor volumes were approximately equal in the four groups. Tumor volume was calculated by the following formula: tumor volume (mm^3^) = 0.5 × (major diameter) × (minor diameter) × (minor diameter). Mice were treated by intraperitoneal injection of 3.0 or 5.0 mg/kg syringolog-1 twice weekly, or intraperitoneal injection of 1.0 mg/kg Btz twice weekly. Control group mice were injected with saline. The level of free light chain in plasma was measured using a Free Light Chain Assay kit (Binding Site, Birmingham, UK). Similarly, six-week-old male NOD/ShiJic-scidJcl mice inoculated with OPM-2/Btz, a Btz resistant cell, were tested.

### Statistical analysis

Analyses were carried out using GraphPad Prism software. Data comparison in multiple groups was carried out by one-way analysis of variance, and the further comparisons between two groups were followed by Dunnett’s pos*t*-test. Two-tailed Student’s *t*-test was performed to compare two groups according to the distribution of data. *P* value of <0.05 was considered statistically significant.
